# Professional Identity Formation and Population Health: a qualitative study of medical students’ experience of Lifestyle Medicine and Prevention

**DOI:** 10.1186/s12909-025-08159-7

**Published:** 2025-11-11

**Authors:** Anna Ogier, Stephen Naulls, Christopher-James Harvey, Ana Baptista, Richard J. Pinder

**Affiliations:** 1 School of Public Health, Imperial College London, London, UK; 2 Imperial College School of Medicine, Imperial College London, London, UK

**Keywords:** Population health, Lifestyle medicine, Undergraduate, Medical, Qualitative, Professional identity formation

## Abstract

**Background:**

Healthcare systems worldwide are facing challenges on multiple fronts: ageing demographics, multimorbidity, staff burnout and persistent health inequalities. There is growing recognition of the need for prevention and population health within education and training programmes. Yet population health has been historically challenging to integrate into clinical-facing curricula.

A novel programme of learning was introduced at Imperial College School of Medicine (London, UK). “Lifestyle Medicine and Prevention” (LMAP) comprises two modules and is a core element of learning across the first two years of undergraduate medical training. In these two modules, medical students explore how lifestyle factors, behaviour and the wider determinants of health influence health outcomes through a population health lens.

**Methods:**

This qualitative study describes how LMAP has impacted the professional identity formation of medical students. In June 2020, 278 students completed an optional, anonymous survey, with 14 students completing in-depth focus groups. Thematic analysis was undertaken of the student responses.

**Results:**

Four key themes were identified regarding the impact of the modules on medical students’ professional identity formation: the wider purpose of a doctor in society, including their role in prevention, the skills required to deliver holistic care, the value of well-being in medical professionals, and the challenges surrounding LMAP’s impact on professional identity.

**Conclusions:**

These results outline how LMAP’s novel approach to incorporating population health teaching for medical students may help address the manifold challenges facing modern healthcare services. By shaping the attitudes of future clinicians to recognise the value of prevention, lifestyle factors and their own personal wellbeing, we are preparing medical students for professional practice in increasingly challenging environments. However, further evaluation is required to evidence the longer-term impact of LMAP on medical students’ professional identity.

**Supplementary Information:**

The online version contains supplementary material available at 10.1186/s12909-025-08159-7.

## Background

Medical school curricula have an important role in shaping students’ professional identity (PI), defined as “a representation of self, achieved in stages over time. resulting in an individual thinking, acting, and feeling like a physician” (p.1446) [1]. Professional identity formation (PIF) is an iterative process involving the “integration of the knowledge, skills, values, and behaviours of a profession with one’s preexisting identity and values” (p.96) [2]. As Jarvis-Selinger et al. outline, PIF occurs at an individual, psychological-level, and at a wider collective level through socialization [3]. This process begins from the start of medical school and continues into students’ clinical careers [4, 5]. PIF has been shown to have a positive impact on student wellbeing [6, 7], and is considered essential to the tenets of medical education as the provision of clinical knowledge [8].

Learners are more likely to act in accordance with the professional values of Medicine, if such values are internalised [3]. Through PIF, learners move from simply demonstrating the expected behaviours of a physician, to being or becoming a physician [9, 10]. Wald highlights the challenge for medical educators to create effective pedagogy to guide learners through this journey, emphasising the role of reflection and socialisation within this [10]. Cruess et al. describe how PIs are not static, with societal expectations of doctors shaped by wider society and evolving healthcare systems [9].

Globally, healthcare systems face multiple, ongoing challenges; under pressure from ageing populations and multi-morbidity [11, 12]. The United Kingdom’s (UK) universal health system (National Health Service, NHS) is no exception [13, 14]. The NHS is increasingly stretched – with rising levels of staff burnout [15, 16], and health inequalities projected to persist [17]. Such challenges prompt calls for strong public health approaches within healthcare [12], with greater emphasis on prevention as outlined in the NHS 10-Year Health Plan [18].

Such shifts in medicine necessitate educational approaches that broaden learners’ understanding of the doctor’s role to incorporate population health. Population health is a broad term, that encompasses “public health medicine and wider disciplines” that “describes the amount of health and its distribution across a population” (p.1) [19]. It incorporates concepts including prevention, the wider determinants of health and health inequalities [20, 21]. Educators should consider how medical curricula can best shape the PI of future clinicians to incorporate population health to meet the wider healthcare challenges.

We introduced a novel programme of learning at Imperial College School of Medicine (London, UK). All 340 + first-year students are introduced to preventive medicine and population health across the first two years of their six-year undergraduate learning: “Lifestyle Medicine and Prevention” (LMAP, pronounced EL-map) [19]. Lifestyle Medicine is a medical discipline that “focuses on the role of lifestyle factors in preventing, managing, and reversing chronic disease” (p.98) [22]. Lifestyle Medicine is a growing discipline with a developing evidence-base [22], however, is not without criticism [23].

The LMAP programme applies a pedagogically informed educational design firmly rooted in the principles of population health. Lifestyle Medicine is used as a vehicle to communicate key topics in population health to medical students [19], including health improvement, social justice and prevention. Educational activities are clinically driven in their content and clinician led. The first year of LMAP covers foundational lifestyle medicine pillars: nutrition, physical activity, sleep, mental health and financial well-being. The second year builds on this, exploring epidemiology, communication skills, life course approaches, global health and health policy.

The two LMAP modules form 15% of the total delivered curriculum across the first two years of the medical degree, comprising approximately 81 teaching contact hours, and were introduced as part of a wider curriculum review in 2019.

LMAP’s three aims can be summarised as following:

1. Build student understanding of the impact of lifestyle factors and behaviours in patients’ lives.
2. Develop student reflection and understanding into their own well-being and behaviours.
3. Embed a population health mindset into the clinical practice of doctors.

LMAP uses a flipped classroom approach: asynchronous self-directed online learning followed by small group, in-person tutorials [24]. Students discuss and reflect how behaviour and lifestyle factors impact health outcomes, facilitated by clinicians and educational faculty, including communication skills teams [19, 25]. Lifestyle Medicine and population health topics are mutually beneficial. For example, for teaching the pillar of sleep, students complete 2-hours online learning on sleep from a population health lens, including its link to non-communicable disease. Students then evaluate sleep interventions in a 1-hour tutorial, considering the impact of wider determinants and reflecting on personal behaviours.

There is emerging consensus on the role and value of Lifestyle Medicine to prevent, manage and even reverse long-term conditions [26, 27], yet less real-world experience of how to mainstream such skills development within medical curricula [19]. Although studies have explored how population health teaching impacts PIF, this has not focussed on undergraduates [28], or is framed by the COVID-19 pandemic [29, 30]. There is limited literature exploring how Lifestyle Medicine impacts undergraduate medical students’ PIF. Yet, requirements for clinicians to adopt population health approaches is well-established. The General Medical Council’s Outcomes for Graduates [31] and the Royal College of Physicians and Surgeons of Canada’s CanMEDS framework [32] emphasize the importance of promoting lifestyle change and advocating for the health of populations and communities.

This paper explores how the introduction of Lifestyle Medicine and population health teaching, through the mechanism of LMAP, at Imperial College School of Medicine, has affected student perceptions of the role of a doctor, and ultimately, how their PI may (or may not) have been influenced.

## Methods

We employed a qualitative methodology to explore how LMAP has influenced, if at all, students’ perceptions of the role of a doctor. The guiding research questions in this study were:

1. *Has LMAP impacted early-year medical students’ perceptions of the role of a doctor?*
2. *If so*,* in what way(s) has LMAP impacted the student perception of the role of a doctor?*
3. *How has this change in perception influenced the professional identity formation of the early-year medical students?*

### Procedure

At Imperial, all first-year undergraduate medical students were invited to complete an optional, online, multi-item survey in June 2020, at the end of their first year of study. The survey lasted approximately 15-minutes and used mixed open and closed-questions to explore the students’ experience of LMAP. This paper will focus on student responses to the specific open-ended question *“Has LMAP changed your perception of what a doctor is/does? Please explain”.* The data pertaining to this specific question has not been published before. However, the responses to a separate question within the same survey are published elsewhere [25].

Additionally, first- and second-year medical students were invited to take part in focus groups that lasted approximately 90-minutes at the end of the academic year in 2021, where attitudes towards LMAP could be explored in greater depth. The focus groups were led by AB, an experienced qualitative researcher, who was not involved in the design or delivery of the LMAP content.

A copy of the survey questions and the questions used in the focus groups are provided as supplementary material.

### Data analysis

Utilising the inductive approach described by Braun and Clarke [33], thematic analysis was undertaken of the text-based survey responses to the question *“Has LMAP changed your perception of what a doctor is/does? Please explain”.* Thematic analysis was also carried out using the same approach for the anonymised transcripts from the focus groups, where students’ responses relating to LMAP and their perception of the role of a doctor were also analysed.

Coder AO conducted the initial review of the survey responses and focus group transcripts. AO familiarised themselves with the data, generated initial codes using an inductive process until no new codes emerged, and identified overarching themes at the semantic level. Coder SN independently familiarised themselves with the data and critically reviewed the codes and themes identified by AO. Coders AO and SN collaboratively discussed the codes and themes, and discrepancies identified were reviewed by coder C-JH, as external auditor. AO and SN refined themes and sub-themes through iterative discussion, using a consensus approach, ensuring codes were grounded in the data, and that each theme was distinct. To validate the process, C-JH independently reviewed a large, random sample of codes across all themes, with any discrepancies resolved through consensus with AO and SN.

To assess whether the student perception of a doctor had altered, AO categorised survey responses as either Yes (perception altered), No (perception unchanged), or N/A (insufficient data). This classification was reviewed by SN and C-JH until consensus agreed.

Analysis was completed utilising NVivo software (QSR International Pty Ltd. (2020) NVivo (released in March 2020) (https://lumivero.com/products/nvivo/).

### Positionality and reflexivity statement

AO and SN are early-career clinicians and medical educators, and part of the LMAP faculty. Although this provides insight into the student learning context, there is risk of unconscious bias, with over-emphasis of themes aligned to their experiences as educators or clinicians [34]. To mitigate this, AO and SN endeavoured to adopt a reflexive stance and recursive nature throughout the analysis, engaging in regular debriefing to critically examine their assumptions, and utilised an inductive approach to ensure themes were grounded in participant accounts.

C-JH is a senior medical education academic, experienced in thematic analysis, and a member of the LMAP faculty. Their experience may risk bias towards unconsciously favouring perspectives aligning with pedagogical frameworks or their professional teaching experiences. Therefore, AO and SN led the analysis, with C-JH offering an alternative, non-clinical perspective, and triangulating findings through their independent review of codes.

### Ethics

Appropriate ethical approval from Imperial College London’s Faculty of Medicine Medical Education Ethics Committee (MEEC1920-181) was granted for this study.

## Results

In the end of year optional survey, out of 364 students in a year group, 278 students consented for their data to be used in this study. 194 students reported a change in perception of a what a doctor is or does, with 29 reporting no change in perception (Table 1). 55 students did not provide a free-text response to this question. 14 students participated in the four focus groups led by AB in June 2021.

**Table 1:** Sample answers for classification of student open-ended responses to the question “Has LMAP changed your perception of what a Doctor is/does? Please explain”. Total survey responses (*n* = 278)

Yes (perception has changed) (*n* = 194)	No (perception has not changed) (*n* = 29)	N/A - not applicable (*n* = 55)
“I now know a doctor not only treats a patient for their condition/presenting complaint but takes into consideration the lifestyle of that individual also” “Yes, doctors can be pioneers for behaviour change in the society.” “Yes. Doctors do not just cure/attempt to ease illness but offer advice on how to prevent illness in the first place.”	“Not really” “It hasn’t changed my perception of a doctor” “It hasn’t”	[blank responses]

Thematic analysis was undertaken on the 223 free text responses in the survey (respondents coded with the prefix SURV-), and the transcripts from the focus groups (respondents coded with the prefix FG-), which were categorised into four main themes, described in the table below (Table 2).

**Table 2:** Themes and sub-themes identified in the thematic analysis

Themes	Sub-themes
1. The doctor’s role in creating resilient and healthy communities (Purpose)	1A – A doctor’s role in prevention 1B – A doctor’s role in population health 1C - Wider roles of a doctor in society
2. Lifestyle Medicine as a means to deliver holistic and person-centred care (Skills)	2A – The value of Lifestyle Medicine for delivering holistic care 2B – The need to empower patients to live healthy lives 2C – The skills that make a good doctor
3. Importance of wellbeing and self-care for medical professionals (Value-add)	3A – Unique challenges of a career in medicine 3B – The importance of good health to be an effective practitioner
4. Resistance and uncertainty (Challenge)	4A – Little to no change in perception of a doctor 4B – Limitations to the role of a doctor

In relation to each theme, we suggest keywords that function as ‘descriptors’ of LMAP’s contribution to students’ perceptions of a doctor’s role: LMAP clarified the *purpose* of the doctor’s role (1), LMAP broadened the perceptions of the *skills* that a doctor should employ (2), LMAP emphasised the *value-add* of well-being as medical professionals (3), and LMAP highlighted *challenges* regarding incorporating population health and lifestyle medicine into a doctor’s role (4).

### Theme 1: Purpose – a doctor’s role in creating resilient and healthy communities

The theme reflects a shift in students’ perception of a doctor’s purpose. Three sub-themes emerged: a doctor’s role in prevention; a doctor’s role in population health; and the wider roles of a doctor in society.

Students’ expectation of a doctor diverged from a traditional focus: from one whose purpose is to treat disease as it arises, towards a role in prevention, tackling the causes of ill-health.

*“Now I understand a doctor’s role is in helping patients to make healthy choices throughout their everyday lives to prevent disease rather than just to treat it when it happens” [SURV-115]*.

*“Going back one step… have you put a plaster over it*,* or have you really tried to fix the wound?” [FG3-PA2]*.

LMAP prompted students to renegotiate their expectations of a doctor’s role, to one that integrates a population-health mindset. Students reflect how clinicians should consider the wider determinants of health and health inequalities within their work.

*“[A doctor’s role is to] improve patients’ health long term - by not only looking at individuals but by looking at whole populations” [SURV-91]*.

*“I know that people who live in deprived areas might have worse health outcomes. But you don’t really ever sit down and think about what the implications of what that means for you as a doctor.” [FG2-PA1]*.

*“A doctor must understand that health varies greatly on the basis of where someone lives*,* even within one city*,* and that even within the NHS*,* services vary.” [SURV-45]*.

Students reported new-found recognition of the wider responsibilities of doctors in society, as educators and counsellors. Students’ identity as future doctors appeared to diversify away from a purely clinical role: to advocate and role model.

*“It expanded my understanding that a doctor is also a teacher: they educate on healthy lifestyles for patients and should act as role models if they can.” [SURV-168]*.

*“A doctor is an advocate in society and accounts for many things other than just medicine… [such as] challenging stigmas and biases in society.” [SURV-41]*.

*“…it’s your responsibility*,* as well as treating patients*,* to advocate [to address] these inequalities… It’s not just all up to politicians or policymakers.” [FG4-PA2]*.

### Theme 2: Skills - Lifestyle Medicine as a means to deliver holistic and person-centred care

This theme denotes students’ perceptions of the modern doctor’s skill set: to deliver holistic, person-centred care. Sub-themes include the value of Lifestyle Medicine to deliver holistic care, a doctor’s role to empower patients, and how Lifestyle Medicine provides important skills for effective doctors.

Students acknowledged a new-found appreciation of the impact of lifestyle on an individual’s health, challenging assumptions that lifestyle advice is simply “common sense”. As a result, students reframe Lifestyle Medicine as an essential clinical tool for delivering holistic care.

*“LMAP has changed my perception of a doctor to someone who can fundamentally alter a patient’s health by [supporting] them to change their lifestyle … A doctor doesn’t need to give the patients medication to ensure their health*,* if he or she can convince the patient to improve their way of life instead.” [SURV-181]*.

*“I believe a doctor should also consider the lifestyle-behaviour [of patients] …implementing a healthier lifestyle can greatly improve a chronic condition” [SURV-206]*.

*“It’s more than common sense as well. … If it’s not taught at medical school*,* future doctors might just assume people know how to be fit and healthy and they just choose not to.” [FG1-PA2]*.

Students also reinterpret the nature of the patient-doctor relationship; to one of a partnership, with doctors using skills in behaviour change and shared-decision making to empower patients. Students emphasise the importance of empathy within this relationship, and to avoid blame.

*“I view the doctor as having a role which serves to guide individuals on their own journey to health rather than providing a quick short-term solution.” [SURV-190]*.

*“There’s much more value to being [in] a partnership… helping the patient decide their own treatment*,* and giving them a little bit of responsibility” [FG1-PA3]*.

*“Yes. I didn’t know doctors played such an integral part in ensuring patients reflected on their behaviours… [to support] positive changes” [SURV-180]*.

*“I now know that it is really important for doctors to always be empathetic*,* as we may not always know what drives unhealthy behaviours and assumptions shouldn’t be made that a person isn’t trying to be healthy” [SURV-91]*.

Indeed, students described how skills gained through LMAP help them to identify what makes a good medical professional, with some feeling emboldened in the type of doctor they hope to be.

*“It really made me realise that yes I’m learning [body systems]*,* I’m learning [biochemistry]. But if I’m not able to coach my patients*,* help my patients improve their health in these lifestyle areas*,* how good a physician am I actually?…*.

*I feel a lot more equipped to help*,* … I feel like I have more in my tool kit as a physician and I just feel so empowered to help people” [FG2-PA5]*.

*“It was almost like I hope that as a doctor I will be able to do this for my patients*,* but then LMAP was teaching me the ways in which I could do that… I came out of it feeling more I guess secure in my image of the kind of doctor that I wanted to be and came out feeling more like that was possible” [FG2-PA1]*.

### Theme 3: Value-add - the importance of wellbeing and self-care of medical professionals

This theme outlines how LMAP has supported students to appreciate the unique challenges that doctors face in their career and the importance of well-being.

Students reflected on how working conditions for doctors were frequently not conducive to wellbeing. Students denoted a shift in their identity expectations, realising that clinicians often lack both knowledge and opportunity to engage in healthy lifestyles.

*“I knew that doctors worked shift hours in hospitals*,* but LMAP helped me realise the impacts that shift working can have” [SURV-71]*.

*“It made me realise that doctors also engage in unhealthy habits and outside of work struggle to manage this*,* hence the increase in binge-drinking/eating on weekends” [SURV-179]*.

*“…Many doctors aren’t educated enough this way themselves” [SURV-84]*.

*“All you ever hear about being a doctor and starting FY1 on nights*,* [is that] it’s going to be awful and you’re going to be working all the time” [FG3-PA1]*.

Students reported how both students and doctors could benefit personally from learning about Lifestyle Medicine and behaviour change. In doing so, students renegotiated their identity expectations, viewing themselves not only as medical students who should prioritise well-being, but also as a future clinician.

*“I never really put my own health as a priority*,* I didn’t really think of it as being part of my role*,* but LMAP has made me realise that my mental and physical well-being will directly impact how I function as a doctor … After all*,* only a doctor who is happy themselves can do well for their patients.” [SURV-160]*.

*“Yes; it has required me to think of doctors as medical students in the future and the idea that we too can easily be patients.” [SURV-161]*.

### Theme 4: Challenge - resistance and uncertainty

The final theme details the challenges associated with LMAP and its impact on PI.

For some students, there was no reported change in their perception. Some students were already familiar with the importance of holistic approaches and LMAP only reinforced these ideas. Students were unable to identify a tangible impact on their development of PI.

*“LMAP definitely shows the importance of dealing with lifestyle when it comes to patient care. But I don’t think it has revealed any duties of a doctor that weren’t already known.” [SURV-112]*.

“*So far*,* I’ve just noticed differences in personal wellbeing. I haven’t been able to relate it with the profession yet*” [SURV-54].

Some students suggested difficulty navigating their professional identity, as they realised doctors had a smaller role in influencing health than first thought. Some questioned the limits of a doctor’s responsibility to provide lifestyle advice.

*“It was hard for me personally to understand how in my everyday role as a doctor what am I going to do about this… I can’t change the system*,* I can’t change these fundamental issues*,* that’s just not within my scope.” [FG2-PA4]*.

*“If a patient came to me and said I’m stressed because of financial issues*,* I’d completely understand [why]… But I didn’t get what I could do about it… Is it even in my place to advise them about something like that?” [FG1-PA1]*.

*“It has made me realize that a lot of the healing/improving one’s health has more to do with their own personal habits and their socioeconomic status than the help of a doctor” [SURV-38]*.

Overall, these results suggest that LMAP has influenced students’ perceptions of a doctor’s role in diverse ways, providing insight into the development of PIF. Many students expanded their PI to encompass population health, Lifestyle Medicine and the importance of well-being. Others have expressed uncertainty regarding the scope of a doctor’s responsibility.

## Discussion

PIF is a “complex, multidimensional, ongoing, and transformative process” (p.96) [2], through which students integrate “the knowledge, skills, values, and behaviours” they perceive of a “competent, humanistic physician” with their own pre-existing identity (p.762) [35]. As a core goal of medical education [10], educators should optimise curricula to support PIF, helping students transition from simply “doing the work of a physician” to “being a physician” (p.1185) [36].

This study demonstrates how LMAP has influenced students’ values and perspectives within their professional identity, preparing future doctors for contemporary healthcare challenges. LMAP has enabled students to assimilate core population health concepts into their understanding of a doctor’s role. It has underscored the value of Lifestyle Medicine for clinical management, and it highlighted the importance of well-being for future clinicians. However, students have also had to renegotiate their expectations of a doctor’s role with the reality of the challenges of the wider clinical environment.

Using the lens of knowledges, skills, value and behaviours [2, 10, 35] we discuss how LMAP has shaped students’ PIF, and the implications for medical educators.

### Knowledge

Integrating population health into the medical curriculum is well-established in the evidence, with medical students often struggling to appreciate its clinical relevance [37–40]. However, Theme 1 demonstrates how LMAP has helped students reinterpret a doctor’s role to encompass core population health concepts – prevention, wider determinants and health inequalities. In the context of an ageing and increasingly co-morbid population [13], it is promising that many students describe a shift from a doctor that is simply a “plaster” to one that tackles the “wound” at its source (*FG3-PA2*). LMAP’s emphasis on prevention has shifted students’ expectations of their professional identity, to one aligned with an active role in promoting health, not simply responding to disease.

Calls for greater emphasis on population health and prevention in medical education are not new [19, 41, 42], and the lack of research within public health education is highlighted [43]. By focusing on understanding fundamental population health concepts, over memorizing facts, we contend that LMAP has helped bridge the historical schism between population health and traditional medical education [38].

### Skills

Theme 2 highlights students’ recognitions of the importance of a doctor who has practical skills in Lifestyle Medicine to achieve better health outcomes. As Lifestyle Medicine is increasingly recognised to effectively manage non-communicable disease [26, 44], there is much to be gained from developing clinicians who consider Lifestyle Medicine foundational to the role of a doctor. Students describe the value of behaviour change and coaching in patient management, viewing Lifestyle Medicine skills as vital components of a “tool kit” for becoming a “good” doctor (FG2-PA5). As the wider literature calls for clinicians skilled in behaviour change to meet wider population health needs [45, 46], LMAP represents a viable means to achieve this.

Teaching Lifestyle Medicine has facilitated the “*transformation of the self into new ways of thinking and relating*” (p.641) [47] through the assimilation of Lifestyle Medicine into a core role of a doctor. This appears to improve students’ self-efficacy [48] as they report feeling secure in the image of the future clinician they hope to be.

Developing self-efficacy in healthcare professionals has a positive impact on PI, which, in turn, can improve resilience and job satisfaction [49, 50]. Indeed, supporting students’ PIF positively impacts student well-being, as they develop a sense of meaning within the profession [6, 51]. Through LMAP - learning about coaching and behaviour change techniques – the students have gained skills in areas that go beyond traditional clinical knowledge, and have developed a more empowered, motivated and well-rounded PI as a result.

### Values

This study also demonstrates how LMAP has prompted students to renegotiate their values, as part of their PIF. Students now identify doctors as potential advocates – who should address health inequalities within society at large. In the same way COVID-19 encouraged students to reconsider the doctor’s role in tackling inequity and social accountability [52], LMAP encourages students to reflect on the broader PI of a doctor in society. Students developed a new perspective of what it means to be a doctor. Crucially, such an identity aligns with the expectations laid out by the UK’s medical regulator, for doctors who engage in “*advocacy for those who are disempowered*” (p.23) [31].

Of note, Lifestyle Medicine has been criticised for attributing too much responsibility on the individual and not the environment [23]. If Lifestyle Medicine fails to account for the impact of individual and environmental factors, it risks exacerbating health inequalities [53]. However, in LMAP, students reflect on their own lifestyles and behaviours in a bid to engender greater empathy about the barriers to behaviour change, and the influence of the wider determinants. By connecting the medical student’s health behaviours with that of the patient and populations, LMAP seeks to reconcile the politically contentious question of agency versus environmental determinism. If understanding the impact of lifestyle factors at both an individual level and a wider population health level is considered a “threshold-concept” (p.850) [54], LMAP demonstrably facilitates this learning process.

### Behaviours

Theme 3 outlines how students developed a new-found understanding of the importance of health behaviours for both personal and professional well-being. Students recognise that doctors are human, and so too can easily “be[come] patients” (SURV-161). Medical students identify strongly as doctors early on in their studies [55], and by reflecting on their wellbeing as students, they are also reflecting on their wellbeing as future doctors. Many junior doctors experience negative effects on their personal life due to challenging work environments, reporting feeling ill-prepared for the reality of being a doctor [56, 57]. Our study demonstrates how LMAP has helped medical students incorporate well-being and self-care into their PI. Fostering well-being in medical students is increasingly recognised as a key objective of medical education [58, 59]. LMAP represents a novel approach to foster positive behaviours through the curriculum, with important implications for work-force resilience.

### Conflict and challenge

Although some students felt empowered through LMAP, others expressed feelings of uncertainty and powerlessness when faced with the constraints of the current health system. PIF can be a frustrating and potentially overwhelming process – as individuals reconcile their current identity with the perceived requirements of the community [60, 61]. Such dissonance can negatively impact a student’s self-efficacy, highlighting the importance of pastoral support and mentorship throughout a student’s PI journey [61].

A minority of students questioned whether it is the job of a doctor to provide lifestyle advice, be it financial or otherwise. Such questions pose an interesting debate as to what should be considered within the scope of a modern medical professional. Although students recognised the importance of a holistic approach to medicine regarding lifestyle factors, where do the limits lie? There is a clear need for discussion and agreement in the UK as to what these limits are. What do we want from our next generation of doctors? In turn, how can we equip medical students to form a professional identity consistent with these values?

Furthermore, we recognise the importance of system-wide change to support the PIF we hope to develop in students [62, 63]. Without such change, students may struggle to apply LMAP principles in practice, which risks undermining the students’ PI. However, the authors argue that teaching LMAP to students at such a formative stage of training [63, 64], represents an important, bottom-up, first step towards this systems-wide change.

### Strengths and limitations

This study benefited from a survey with a high response rate, and in-depth discussion through focus groups. However, as the questionnaire and focus groups were completed as part of an evaluative research study, there is a risk of response bias. As the study was carried out in a single, undergraduate medical institution based in London, United Kingdom, this limits generalisability. As the participants were in their first two years of medical education, their LMAP experiences were relatively recent. This study could not demonstrate change to practice or behaviour, beyond those suggested by students’ self-report.

Although LMAP is only taught in the first two-years of the programme, population health is consolidated as a theme across the six-years of the curriculum. However, it remains to be seen what the long-term impact is - whether LMAP changes the future practice or thinking of medical professionals. Further evaluation is underway to explore this further.

The authors acknowledge that PIF is influenced by multiple factors, which can be difficult to isolate from the formal LMAP curriculum. This includes internal factors such as students’ personal circumstances, and external factors, such as the hidden curriculum and the wider learning environment [4, 65]. PIF is a socially constructed process, influenced by socialisation [63]. In LMAP, much of the learning occurs during small group reflections within tutorials. This process is likely influenced by the hidden curriculum; as students both consciously and unconsciously share implicit learning and expectations regarding their PIs [47, 66]. The hidden curriculum can both help and hinder the PIF of medical students [66, 67] and although not within the scope of this manuscript, its influence warrants further critical exploration [67].

### Implications

Introducing LMAP has been challenging but arguably beneficial for students. However, embedding it as part of a busy curriculum requires buy-in from senior leadership. Because Lifestyle Medicine is a new and interdisciplinary field, teaching staff also need proper support to deliver learning effectively. The LMAP team is now sharing the lessons learned from local experience to support wider roll-out in other institutions.

## Conclusions

In conclusion, this paper presents a novel curriculum development that can potentially address a variety of pressing healthcare challenges. To mitigate the rising tide of long-term conditions, modern medicine demands clinicians who are instilled with a population health mindset and are appropriately trained to manage lifestyle factors. Furthermore, amid spiralling levels of mental ill-health among younger people, our health systems need resilient and healthy workers.

Incorporating Lifestyle Medicine and Population Health learning into undergraduate medical education has had an important role in shaping and broadening medical students’ “knowledge, skills, values and behaviours” (p.96) [2]: impacts that might be better prepare them for the challenging working conditions of the healthcare system.

We propose that LMAP and its constituent elements are a potentially transformative approach to medical education through its impact on PIF. Medical schools worldwide should consider Lifestyle Medicine and Prevention as a means of delivering preventive medicine and population health to the healthcare leaders of the future.

## Supplementary Information

Supplementary Material 1.

Supplementary Material 2.

## Data Availability

The datasets used and analysed during the current study are available from the corresponding author on reasonable request.

## Abbreviations

LMAP: Lifestyle Medicine and Prevention
NHS: National Health Service
UK: United Kingdom
PI: Professional Identity
PIF: Professional Identity Formation

## References

[1] Cruess RL, Cruess SR, Boudreau JD, Snell L, Steinert Y. Reframing medical education to support professional identity formation. Acad Med. 2014;89(11):1446–51. 10.1097/acm.0000000000000427. 25054423 10.1097/ACM.0000000000000427

[2] Mount GR, Kahlke R, Melton J, Varpio L. A critical review of professional identity formation interventions in medical education. Acad Med. 2022;97(11S):96–106. 10.1097/acm.0000000000004904. 10.1097/ACM.0000000000004904 35947478

[3] Jarvis-Selinger S, Macneil KA, Costello GRL, Lee K, Holmes CL. Understanding professional identity formation in early clerkship: a novel framework. Acad Med. 2019;94(10):1574–80. 10.1097/acm.0000000000002835. 31192797 10.1097/ACM.0000000000002835

[4] Findyartini A, Greviana N, Felaza E, Faruqi M, Zahratul Afifah T, Auliya Firdausy M. Professional identity formation of medical students: a mixed-methods study in a hierarchical and collectivist culture. BMC Med Educ. 2022;22(1):443. 10.1186/s12909-022-03393-9. 35676696 10.1186/s12909-022-03393-9 PMC9175156

[5] Krishnasamy N, Hasamnis AA, Patil SS. Developing professional identity among undergraduate medical students in a competency-based curriculum: educators’ perspective. J Educ Health Promot. 2022;11(1):361. 10.4103/jehp.jehp_329_22. 36618475 10.4103/jehp.jehp_329_22 PMC9818684

[6] Toubassi D, Schenker C, Roberts M, Forte M. Professional identity formation: linking meaning to well-being. Adv Health Sci Educ Theory Pract. 2023;28(1):305–18. 10.1007/s10459-022-10146-2. 35913664 10.1007/s10459-022-10146-2 PMC9341156

[7] McNeill KG, Kerr A, Mavor KI. Identity and norms: the role of group membership in medical student wellbeing. Perspect Med Educ. 2013;3(2):101–12. 10.1007/s40037-013-0102-z. 10.1007/s40037-013-0102-z PMC3976477 24347246

[8] Wilson I, Cowin LS, Johnson M, Young H. Professional identity in medical students: pedagogical challenges to medical education. Teach Learn Med. 2013;25(4):369–73. 10.1080/10401334.2013.827968. 24112208 10.1080/10401334.2013.827968

[9] Cruess RL, Cruess SR, Steinert Y. Amending miller’s pyramid to include professional identity formation. Acad Med. 2016;91(2):180–5. 10.1097/acm.0000000000000913. 26332429 10.1097/ACM.0000000000000913

[10] Wald HS. Professional identity (Trans)formation in medical education. Acad Med. 2015;90(6):701–6. 10.1097/acm.0000000000000731. 25881651 10.1097/ACM.0000000000000731

[11] World Health Organization. World report on ageing and health. World Health Organization; 2015; p. 9789241565042. Report No.

[12] World Health Organization Regional Office for Europe. Facing the future: opportunities and challenges for 21st-century public health in implementing the sustainable development goals and the health 2020 policy framework. Copenhagen: World Health Organization. Regional Office for Europe.; 2018. Report No.: WHO/EURO:2018-2244-41999-57728.

[13] Kingston A, Robinson L, Booth H, Knapp M, Jagger C. Projections of multi-morbidity in the older population in England to 2035: estimates from the population ageing and care simulation (PACSim) model. Age Ageing. 2018;47(3):374–80. 10.1093/ageing/afx201. 29370339 10.1093/ageing/afx201 PMC5920286

[14] Nicol E. The ageing population in healthcare: a challenge to, and in, the workforce. Clin Med. 2017;17(4):291–2. 10.7861/clinmedicine.17-4-291. 10.7861/clinmedicine.17-4-291 PMC6297645 28765401

[15] Gemine R, Davies GR, Tarrant S, Davies RM, James M, Lewis K. Original research: Factors associated with work-related burnout in NHS staff during COVID-19: a cross-sectional mixed methods study. BMJ Open. 2021;11(1):e042591. Available from: https://www.ncbi.nlm.nih.gov/pmc/articles/PMC7844932/:10.1136/bmjopen-2020-042591. 10.1136/bmjopen-2020-042591 PMC7844932 33509850

[16] Health and Social Care Committee. Workforce burnout and resilience in the NHS and Social Care: UK Parliament; 2021 [Last updated: 08/06/2021]. Available from: https://publications.parliament.uk/pa/cm5802/cmselect/cmhealth/22/2202.htm. Accessed: 10/06/2024.

[17] Raymond AWT, Douglas HR, Head A, Kypridemos C, Rachet-Jacquet L. Health inequalities 2040: current and projected patterns of illness by level of deprivation in England: The Health Foundation; 2024. Available from: https://www.health.org.uk/publications/health-inequalities-in-2040. Accessed: 12/06/2024.

[18] Department of Health and Social Care. 10 Year Health Plan for England: fit for the future 2025 [Last updated 30/07/2025]. Available from: https://www.gov.uk/government/publications/10-year-health-plan-for-england-fit-for-the-future. Accessed 01/09/2025.

[19] Pinder R, Bauld L, Findlater H, Mohanamurali A, Johnson A, Birrell F. Is it time to embed lifestyle medicine in undergraduate and postgraduate curricula? Lifestyle Med. 2022. 10.1002/lim2.59.

[20] Institute of Medicine. The future of the public’s health in the 21st century. Washington, DC: National Academies; 2003. p. 536.

[21] Kindig D. What is population health? Am J Public Health. 2011;93(3):380–3. 10.2105/AJPH.93.3.380. 10.2105/ajph.93.3.380 PMC1447747 12604476

[22] Lippman D, Stump M, Veazey E, Guimarães ST, Rosenfeld R, Kelly JH et al. Foundations of Lifestyle Medicine and its Evolution. Mayo Clinic Proceedings: Innovations, Quality & Outcomes. 2024;8(1):97-111.10.1016/j.mayocpiqo.2023.11.004. 10.1016/j.mayocpiqo.2023.11.004 PMC10831813 38304165

[23] Nunan D, Blane DN, McCartney M. Exemplary medical care or Trojan horse? An analysis of the ‘lifestyle medicine’ movement. Br J Gen Pract. 2021;71(706):229–32. 10.3399/bjgp21X715721. 33926883 10.3399/bjgp21X715721 PMC8087320

[24] Hew KF, Lo CK. Flipped classroom improves student learning in health professions education: a meta-analysis. BMC Med Educ. 2018;18:38. 10.1186/s12909-018-1144-z. 29544495 10.1186/s12909-018-1144-z PMC5855972

[25] Harvey C-J, Maile EJ, Baptista A, Pinder RJ. Teaching and learning lifestyle medicine during COVID-19: how has living during a pandemic influenced students’ understanding and attitudes to self-care and population health? A qualitative analysis. BMC Med Educ. 2022;22:532. 10.1186/s12909-022-03590-6. 35804335 10.1186/s12909-022-03590-6 PMC9270827

[26] Sadiq IZ. Lifestyle medicine as a modality for prevention and management of chronic diseases. J Taibah Univ Med Sci. 2023;18(5):1115–7. 10.1016/j.jtumed.2023.04.001. 37187803 10.1016/j.jtumed.2023.04.001 PMC10176046

[27] National Institute for Health and Care Excellence. Type 2 diabetes: prevention in people at high risk 2012. Updated 15 Sep 2017. Available from: https://www.nice.org.uk/guidance/ph38. Accessed: 20/06/2024.

[28] Biehl V, Bänziger A, Wieber F. A longitudinal study on the professional identity formation of health promotion practitioners: evidence from undergraduate students in Switzerland. Front Med. 2025;12:103389fmed20251491467. 10.3389/fmed.2025.1491467 PMC11848852 39995689

[29] Stetson GV, Dhaliwal G. Using a time out: reimagining professional identity formation after the pandemic. Med Educ. 2021;55(1):131–4. 10.1111/medu.14386. 33030782 10.1111/medu.14386 PMC7675445

[30] Luman AA, Bagley M, Colbert-Getz JM, Christensen T, Lindsley JE, Chow CJ. Am i even a med-student anymore? A mixed-methods study of the impact of the initial disruptions caused by the COVID-19 pandemic on medical student professional identity formation. Med Sci Educ. 2022;32(6):1387–95. 10.1007/s40670-022-01652-4. 36277267 10.1007/s40670-022-01652-4 PMC9579630

[31] General Medical Council. Outcomes for Graduates 2018 [Last updated 25/02/2020]. Available from: https://www.gmc-uk.org/education/standards-guidance-and-curricula/standards-and-outcomes/outcomes-for-graduates/outcomes-for-graduates. Accessed: 20/01/2025.

[32] Royal College of Physicians and Surgeons of Canada. The CanMEDS Framework 2015. Available from: https://www.royalcollege.ca/en/standards-and-accreditation/canmeds.html. Accessed 01/09/2025.

[33] Braun V, Clarke V. Using thematic analysis in psychology. Qual Res Psychol. 2006;3(2):77–101. 10.1191/1478088706qp063oa.

[34] Braun V, Clarke V. Reflecting on reflexive thematic analysis. Qualitative research in sport Exerc Health. 2019;11(4):589–97. 10.1080/2159676X.2019.1628806.

[35] Holden MD, Buck E, Luk J, Ambriz F, Boisaubin EV, Clark MA, et al. Professional identity formation. Acad Med. 2015;90(6):761–7. 10.1097/acm.0000000000000719. 25853688 10.1097/ACM.0000000000000719

[36] Jarvis-Selinger S, Pratt DD, Regehr G. Competency is not enough. Acad Med. 2012;87(9):1185–90. 10.1097/acm.0b013e3182604968. 22836834 10.1097/ACM.0b013e3182604968

[37] Mahoney JF, Fox MD, Chheda SG. Overcoming challenges to integrating public and population health into medical curricula. Am J Prev Med. 2011;41(4):170–5. 10.1016/j.amepre.2011.06.025. 10.1016/j.amepre.2011.06.025 21961660

[38] Ruis A, Golden RN. The schism between medical and public health education: a historical perspective. Acad Medicine: J Association Am Med Colleges. 2008;83(12):1153–7. 10.1097/acm.0b013e31818c6583. 10.1097/ACM.0b013e31818c6583 19202484

[39] Ben-Shlomo Y. Public health education for medical students: reflections over the last two decades. J Public Health. 2010;32(1):132–3. 10.1093/pubmed/fdp111. 10.1093/pubmed/fdp111 19959495

[40] Finkel ML. Integrating the public health component into the medical school curriculum. Public Health Reports®. 2012;127(2):145–6. 10.1177/003335491212700201. 22379212 10.1177/003335491212700201 PMC3268797

[41] AbdulRaheem Y. Unveiling the significance and challenges of integrating prevention levels in healthcare practice. J Prim Care Community Health. 2023;14(14). 10.1177/21501319231186500. 10.1177/21501319231186500 PMC10350749 37449436

[42] Maeshiro R, Johnson I, Koo D, Parboosingh J, Carney JK, Gesundheit N, et al. Medical education for a healthier population: reflections on the Flexner report from a public health perspective. Acad Med. 2010;85(2):211–9. 10.1097/acm.0b013e3181c885d8. 20107345 10.1097/ACM.0b013e3181c885d8

[43] Gillam S, Maudsley G. Public health education for medical students: rising to the professional challenge. J Public Health. 2010;32(1):125–31. 10.1093/pubmed/fdp108. 10.1093/pubmed/fdp108 19959496

[44] Richardson K, Petukhova R, Hughes S, Pitt J, Yücel M, Segrave R. The acceptability of lifestyle medicine for the treatment of mental illness: perspectives of people with and without lived experience of mental illness. BMC Public Health. 2024;24(1):171. 10.1186/s12889-024-17683-y. 38218774 10.1186/s12889-024-17683-y PMC10787508

[45] Grant C, Jopling H. Health coaching: a necessary role for medical students? Public Health. 2021;190:52–4. 10.1016/j.puhe.2020.11.001. 33340920 10.1016/j.puhe.2020.11.001

[46] Maini A, Fyfe M, Kumar S. Medical students as health coaches: adding value for patients and students. BMC Med Educ. 2020;20(1):182. 10.1186/s12909-020-02096-3. 32493308 10.1186/s12909-020-02096-3 PMC7271500

[47] Goldie J. The formation of professional identity in medical students: considerations for educators. Med Teach. 2012;34(9):641–8. 10.3109/0142159x.2012.687476. 10.3109/0142159X.2012.687476 22905665

[48] Turan S, Valcke M, Aper L, Koole S, Derese A. Studying self-efficacy beliefs in medical education. Procedia - Social Behav Sci. 2013;93:1311–4. 10.1016/j.sbspro.2013.10.034.

[49] Mei XX, Wang HY, Wu XN, Wu JY, Lu YZ, Ye ZJ. Self-efficacy and professional identity among freshmen nursing students: a latent profile and moderated mediation analysis. Front Psychol. 2022. 10.3389/fpsyg.2022.779986. 10.3389/fpsyg.2022.779986 PMC8927723 35310284

[50] Yi R, Zhou Z, Ma W, Yang C, Wang F, Wu J. Mediating role of psychological resilience in the relationship between self-efficacy and professional identity among nurses. Biotechnol Genet Eng Rev. 2023;40(2):726–38. 10.1080/02648725.2023.2190943. 36966473 10.1080/02648725.2023.2190943

[51] Chandran L, Iuli RJ, Strano-Paul L, Post SG. Developing a way of being: deliberate approaches to professional identity formation in medical education. Acad Psychiatry. 2019;43(5):521–7. 10.1007/s40596-019-01048-4. 30993596 10.1007/s40596-019-01048-4

[52] Moula Z, Horsburgh J, Scott K, Rozier-Hope T, Kumar S. The impact of Covid-19 on professional identity formation: an international qualitative study of medical students’ reflective entries in a global creative competition. BMC Med Educ. 2022;22:545. 10.1186/s12909-022-03595-1. 35836173 10.1186/s12909-022-03595-1 PMC9282904

[53] Duplantier SC, Barach R, Emmert-Aronson SJS, Markle B. EA. Equitable Access to Lifestyle Medicine: FQHCs, YMCAs, Trauma-Informed Health Coaching, and Community as Medicine. American Journal of Lifestyle Medicine. 2025.10.1177/15598276251325799 10.1177/15598276251325799 PMC11920983 40114668

[54] Neve H, Wearn A, Collett T. What are threshold concepts and how can they inform medical education? Med Teach. 2016;38(8):850–3. 10.3109/0142159x.2015.1112889. 26609736 10.3109/0142159X.2015.1112889

[55] Burford B, Rosenthal-Stott HES. First and second year medical students identify and self-stereotype more as doctors than as students: a questionnaire study. BMC Med Educ. 2017;17:209. 10.1186/s12909-017-1049-2. 29132332 10.1186/s12909-017-1049-2 PMC5683566

[56] Goodyear HM. First year doctors experience of work related wellbeing and implications for educational provision. Int J Med Educ. 2014;5:103–9. 10.5116/ijme.5380.6ef1. 25341219 10.5116/ijme.5380.6ef1 PMC4207178

[57] Monrouxe LV, Bullock A, Gormley G, Kaufhold K, Kelly N, Roberts CE, et al. New graduate doctors’ preparedness for practice: a multistakeholder, multicentre narrative study. BMJ Open. 2018;8(8):e023146. 10.1136/bmjopen-2018-023146. 10.1136/bmjopen-2018-023146 PMC6119440 30158236

[58] Kushner RF, Kessler S, McGaghie WC. Using behavior change plans to improve medical student self-care. Acad Med. 2011;86(7):901–6. 10.1097/acm.0b013e31821da193. 21617509 10.1097/ACM.0b013e31821da193 PMC3128665

[59] Bull S, Danso-Bamfo S, Ogden J, Kumar S. Navigating the boundaries of health and identity: medical students’ perspectives. Clin Teach. 2025. 10.1111/tct.70028. 10.1111/tct.70028 PMC11742921 39827899

[60] Frost HD, Regehr G. I am a doctor: negotiating the discourses of standardization and diversity in professional identity construction. Acad Med. 2013;88(10):1570. 10.1097/acm.0b013e3182a34b05. 23969361 10.1097/ACM.0b013e3182a34b05

[61] Sarraf-Yazdi S, Goh S, Krishna L. Conceptualizing professional identity formation in medicine. Acad Med. 2024;99(3):343–343. 10.1097/acm.0000000000005559. 38015999 10.1097/ACM.0000000000005559

[62] Wong A, Trollope-Kumar K. Reflections: an inquiry into medical students’ professional identity formation. Med Educ. 2014;48(5):489–501. 10.1111/medu.12382. 24712934 10.1111/medu.12382

[63] Cruess RL, Cruess SR, Boudreau JD, Snell L, Steinert Y. A schematic representation of the professional identity formation and socialization of medical students and residents. Acad Med. 2015;90(6):718–25. 10.1097/acm.0000000000000700. 25785682 10.1097/ACM.0000000000000700

[64] Tagawa M. Scales to evaluate developmental stage and professional identity formation in medical students, residents, and experienced doctors. BMC Med Educ. 2020. 10.1186/s12909-020-1942-y. 10.1186/s12909-020-1942-y PMC7011234 32041597

[65] Lesser CS. A behavioral and systems view of professionalism. JAMA. 2010;304(24):2732. 10.1001/jama.2010.1864. 21177508 10.1001/jama.2010.1864

[66] Scholtens S, Barnhoorn P, Fleer J. Education to support professional identity formation in medical students: guiding implicit social learning. Int J Med Educ. 2023;14:19–22. 10.5116/ijme.63f3.ddcb. 36869864 10.5116/ijme.63f3.ddcb PMC10693397

[67] Brown MEL, Coker O, Heybourne A, Finn GM. Exploring the hidden curriculum’s impact on medical students: Professionalism, identity formation and the need for transparency. Med Sci Educ. 2020;30(3):1107–21. 10.1007/s40670-020-01021-z. 34457773 10.1007/s40670-020-01021-z PMC8368648

