# Supplementary material for: Professional Identity Formation and Population Health: a qualitative study of medical students’ experience of Lifestyle Medicine and Prevention
Source: BMC Med Educ. 2025 Nov 11;25:1576. doi: 10.1186/s12909-025-08159-7 (PMC12607180; doi:10.1186/s12909-025-08159-7)
Supplement: Supplementary file 1 — Supplementary Material 1. [file 12909_2025_8159_MOESM1_ESM.docx]

**SUPPLEMENTARY MATERIALS – A**

**LMAP End of Year Online Survey**

Lifestyle Medicine & Prevention 1 (LMAP) is a new Phase 1a module, intended to develop a number of skills required of doctors. We would like to capture your experiences of the LMAP, so that we can learn from them. Your views will be of great value to the educational team in understanding and enhancing what LMAP can provide.

**This survey is anonymous and your answers cannot be linked to you.** Participation in this survey is voluntary. Your academic progression will not be affected in any way by your decision about whether or not to participate. If you choose to participate, your anonymous data may be used in an aggregate form. Completing this survey will be taken as presumed consent for your answers to be used for evaluation and research purposes.

If you choose to participate in this survey, you will be asked a series of questions. Please think about your personal experiences, and answer the questions honestly. **It will take you around 10-15 minutes to complete the survey.**

*For full details about the evaluative research, which has ethical approval from the Medical Education Ethics Committee of Imperial College London (MEEC1920-181), please see the relevant sections/information in*[*this comprehensive information sheet*](https://imperiallondon-my.sharepoint.com/:b:/g/personal/amadeira_ic_ac_uk/EZ_WVq68U6hEjq7BncXt56wBCzvTBhktxXqrw7dijlwO5w?e=1KIJ8S)*. If you have any questions please contact the Principal Investigator - Dr Ana Baptista (EMAIL ADDRESS). Ana is a Principal Teaching Fellow and member of MERU - Medical Education Research Unit, in the Faculty of Medicine. One of Ana's roles is to evaluate the changes made to your medical degree in collaboration with module and domain leads and the educational teams. Ana does not teach nor assess in any modules of MBBS.*

Thank you very much for your collaboration!

The purpose of this survey is to help you reflect on your LMAP learning. This is primarily to help us improve the course but we may want to use this data for the purposes of research. Please indicate below if you are happy for your data to be used in this way

- Yes I consent to my anonymised data being used for the purposes of research
- No I do not consent to my anonymised data being used for the purposes of research

Which topic group were you in for the last term of teaching?

- Sleep
- Mental Health
- Physical Activity
- Nutrition
- Money and Well-being

How did you decide your order of preference for the student choice component options?

[Open text response]

How would you describe your experience of LMAP teaching so far, considering both the positive and negative aspects of the course?

[Open text response]

What concept(s) taught in LMAP did you find the most challenging or difficult to grasp? Please explain.

[Open text response]

Did you resolve the difficulty you mentioned above? If so, how?

[Open text response]

In what ways has LMAP challenged or altered your understanding of medicine and health (if at all)?

[Open text response]

Has LMAP changed your perception of what a doctor is / does? Please explain.

[Open text response]

Has your LMAP learning encouraged you to change any of your health behaviours?

Please explain how it has or has not impacted your health behaviours.

[Open text response]

Has the experience of COVID-19 changed the importance you place on Lifestyle Medicine? If so, how?

[Open text response]

*For the below questions please indicate the extent to which you agree or disagree with the below statements. Your honest answer is the best answer, and usually your first instinct is the most truthful.*

I found the content of LMAP in Terms 1 and 2 - that is the online learning, tutorials and communication skills - interesting:

- Strongly agree
- Somewhat agree
- Neither agree or disagree
- Somewhat disagree
- Strongly disagree

I found the content of Term 3 LMAP - that is the choice stream and assessment - interesting:

- Strongly agree
- Somewhat agree
- Neither agree or disagree
- Somewhat disagree
- Strongly disagree

I recognise the relevance of LMAP to my future role as a doctor.

- Strongly agree
- Somewhat agree
- Neither agree or disagree
- Somewhat disagree
- Strongly disagree

I found LMAP (including the student choice component), to be well-organised:

- Strongly agree
- Somewhat agree
- Neither agree or disagree
- Somewhat disagree
- Strongly disagree

We would like to know whether different student groups have different perspectives, so that we can work towards a more inclusive learning experience. Please skip any questions you do not feel comfortable answering. Your answers are anonymous and cannot be traced back to you.

What is your age?

[Open text response]

Do you have a previous higher education degree?

- Yes
- No

What gender do you identify as?

- Male
- Female
- Other
- Prefer not to say

Would you identify yourself as being a member of an ethnic minority at Imperial College?

- Yes
- No
- Prefer not to say

What is your ethnicity?

- English / Welsh/ Scottish/ Northern Irish/ British
- Irish
- Gypsy or Irish Traveller
- Any other White background
- White and Black Caribbean
- White and Black African
- White and Asian
- Any other Mixed/ Multiple ethnic background
- Indian
- Pakistani
- Bangladeshi
- Chinese
- Any other Asian background
- African
- Caribbean
- Any other Black/ African/ Caribbean background
- Arab
- Any other ethnic group
- Prefer not to say

Would you identify yourself as being from a social background where few people attend university?

- Yes
- No
- Prefer not to say

We thank you for your time spent taking this survey.

Your response has been recorded.
