# Supplementary material for: Professional Identity Formation and Population Health: a qualitative study of medical students’ experience of Lifestyle Medicine and Prevention
Source: BMC Med Educ. 2025 Nov 11;25:1576. doi: 10.1186/s12909-025-08159-7 (PMC12607180; doi:10.1186/s12909-025-08159-7)
Supplement: Supplementary file 2 — Supplementary Material 2. [file 12909_2025_8159_MOESM2_ESM.docx]

**Supplementary Material - B**

**Focus Groups – Questions**

**Do please think about your experience across the two years. Think about your experience of LMAP as whole.**

1. What did you expect lifestyle medicine (as a discipline) to be when you started University?

2. Has that changed? And how?

**[From this point on, focus on LMAP as module – whole LMAP experience re Phase 1a and 1b]**

3. How do you feel about LMAP from first year compared to the second year?
(Taking more seriously? More valued? More interesting themes?)

4. What part of LMAP (both 1a and 1b; in general) was your favourite and why?

/ What was your favourite topic of LMAP 1a AND sub-units within LMAP 1b? And why?

5. What concepts in LMAP did you find really challenging or difficult to grasp? And how did you go about solving them?

5.1. To what extent would you change how those concepts were taught?

6. In what ways has LMAP changed (or challenged) your understanding of medicine and health (if at all)?

7. Has LMAP changed your perception of what a doctor is / does? How/Why?

8. Has your experience of LMAP had an impact on you personally? How/Why?

/ Do you feel LMAP has made any difference to your own well-being and how you took/have been taking care of yourselves?

(Follow up if needed)

8.1. How has LMAP learning encouraged you to change any of your health behaviours? Why?

**Let’s now focus on the delivery of T&L and assessment**

9. You experienced 2 different types of summative assessments: 1A podcast and the 1B summative exam. What was your experience of those types of summative assessments and how these affected your learning experience/ how you learnt? And the knowledge you acquired and consolidated about LMAP?

10. How would you describe your experience of LMAP teaching focusing on Phase 1a, considering both the positive and negative aspects of the course?

10.1. What particular topic block did you choose for your (student) choice component? And why?

10.2. In Phase 1a, what did they think of the footage with the actors – cheesy, impactful, realistic, relatable/believable? Value for their learning experience?

10.3. How did you manage the pre-reading and time management?

11. How would you describe your experience of LMAP teaching focusing on Phase 1b, considering both the positive and negative aspects of the course?

11.1. What do you feel about the balance of GOLs to LOLs?

11.2. Did you use the active podcasts? Did you value that? Why?

(11.3. What do you think is the usefulness of the structured format of the student facing question/discussion and how did you think this impacted on your learning within LMAP?)

12. Looking back, what would you have more of? And what would you get rid of/ or less of? Content/themes and activities/delivery aspects?

/ Are there any topics that you would like to have covered? (but weren’t)
